# Integrative analysis of miRNAs-mRNAs reveals that miR-182 up-regulation contributes to proliferation and invasion of nasopharyngeal carcinoma by targeting PTEN

**DOI:** 10.18632/aging.103316

**Published:** 2020-06-15

**Authors:** Zhaohui Shi, Rushi Wang, Ligui Huang, Xiaodong Chen, Min Xu, Dingjun Zha, Yanhong Ma

**Affiliations:** 1 Department of Otorhinolaryngology Head and Neck Surgery, Air Force Medical University, XiJing Hospital, Xian, Shanxi, China; 2 Department of Otolaryngology Head and Neck Surgery, The Third Xiangya Hospital of Central South University, Changsha, Hunan, China; 3 Department of Otorhinolaryngology Head and Neck Surgery, The 908th Hospital of Chinese People’s Liberation Army Joint Logistic Support Force, Nanchang, Jiangxi, China

**Keywords:** miR-182, PTEN, nasopharyngeal carcinoma, proliferation, invasion

## Abstract

Objective: Several miRNAs have been found to be abnormally expressed during nasopharyngeal carcinoma development. Nevertheless, the interaction between miRNAs and downstream genes remains unexploited. In this study, we aim to investigate miRNAs-mRNAs interaction and the mechanism of miR-182 in NPC.

Results: Integrative analysis identified several hub-miRNAs that drive NPC pathogenesis. The expression of miR-182 was notably increased in 32 NPC tissues and cell lines (CNE1 and 5-8F). Up-regulation of miR-182 was strongly correlated with poor prognosis of NPC patients. Moreover, the proliferation and invasion of NPC cells were notably increased in miR-182 mimics condition and decreased in miR-182 inhibitor condition. Furthermore, PTEN was verified to be a target of miR-182 and overexpression of PTEN could abrogate the promotion effect of miR-182 mimics on NPC invasion.

Conclusions: We identified several hub-miRNAs that may drive NPC pathogenesis. MiR-182 could promote proliferation and invasion of NPC cells via targeting PTEN, which provides a new insight into the clinical therapy of NPC.

Materials and Methods: Genome-wide miRNAs of NPC tissues was analyzed using high-throughput sequencing and bioinformatics tools. QRT-PCR experiment was conducted to measure relative expression level. Dual-luciferase reporter assay was used to verify target relationship. The proliferation and invasion of transfected cells were measured by CCK-8 and transwell assay.

## INTRODUCTION

Nasopharyngeal carcinoma (NPC) is a malignant cancer frequently seen in Southeast Asia [1, 2]. NPC is derived from epithelial cells and associated with complex biochemical, physiological and molecular modification. Although therapeutic strategy for NPC has been improved in recent years, the survival rate of NPC patients still unsatisfactory because of distant metastasis [3]. Therefore, it is important to investigate the molecular mechanisms underlying NPC development to find potential prognostic biomarkers for NPC.

MicroRNAs (miRNAs) are a family of small, noncoding RNAs that regulate gene expression via binding to the 3’untranslational region (3’UTR) of mRNAs [4, 5]. Aberrant expression of miRNAs was observed in various malignant tumor, for example breast cancer, gliomas and lung cancer [6–8]. Previous studies suggested that miRNAs play crucial roles in cellular differentiation, proliferation and apoptosis [9]. MiR-182 is a cancer-related oncogenic miRNA and dysregulated in a variety of human diseases [10, 11], for example, miR-182 could promote proliferation and invasion of mesothelioma cells via targeting FOXO1 [12]. However, the role of miR-182 in NPC remains largely unknown. Phosphatase and tensin homologue (PTEN) was discovered as a tumor suppressor and its decreased expression was observed in NPC [13, 14].

In this study, we identified differentially expressed miRNAs in NPC by means of miRNA-seq and bioinformatics tools. Subsequently, we performed miRNAs-mRNAs integrative analysis by integrating mRNAs expression dataset from GEO database. Several hub-miRNAs that may drive NPC pathogenesis were identified. Moreover, we focused on miR-182 and further studies revealed that miR-182 could promote proliferation and invasion of NPC cells by targeting PTEN, which provides a new insight into the treatment of NPC.

## RESULTS

### Overall expression of miRNAs in NPC

**Figure 1:**
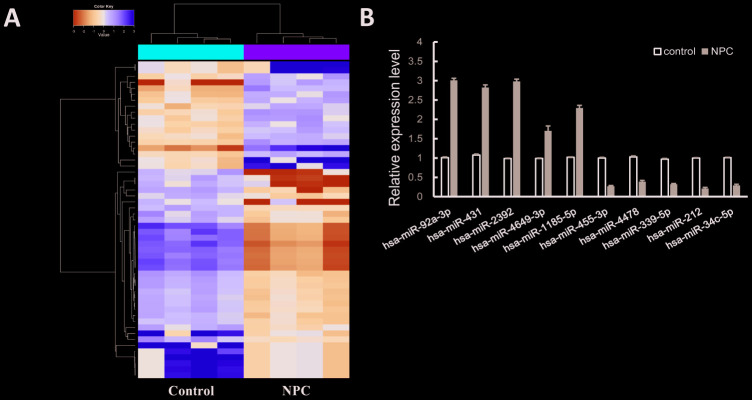
**MiRNAs expression profile in NPC.** (**A**) Heatmap of the differentially expressed miRNAs. Yellow for the up-regulated miRNAs and blue for down-regulated miRNAs. (**B**) QRT-PCR validation of 10 randomly selected miRNAs.

A total of 102 differentially expressed miRNAs were identified in NPC samples by bioinformatics tools, including 39 up-regulated miRNAs and 63 down-regulated miRNAs (|FC| > 1.5, *P*-value < 0.05). The hierarchical cluster analysis revealed evident expression pattern of abnormally expressed miRNAs between NPC and control group (Figure 1A). Top 20 dysregulated miRNAs with significant expression changes was listed in Table 1. To verify the reliability of sequencing data, we randomly selected 10 miRNAs (up-regulated: hsa-miR-92a-3p, hsa-miR-431, hsa-miR-2392, hsa-miR-4649-3p, hsa-miR-1185-5p; down-regulated: hsa-miR-212, hsa-miR-34c-5p, hsa-miR-455-3p, hsa-miR-4478, hsa-miR-339-5p) to measure their expression by qRT-PCR experiment. The results were consistent with sequencing data (Figure 1B).

**Table 1:** Top 20 differentially expressed miRNAs (*P* < 0.05).

**Regulation**	**miRNAs**
Up-regulated:	hsa-miR-182-5p; hsa-miR-92a-3p; hsa-miR-431; hsa-miR-2392; hsa-miR-452; hsa-miR-132-5p; hsa-miR-1185-5p; hsa-miR-36; hsa-miR-1183; hsa-miR-4649-3p
Down-regulated:	hsa-miR-1234-5p; hsa-miR-32-3p; hsa-miR-212; hsa-miR-34c-5p; hsa-miR-4478; hsa-miR-455-3p; hsa-miR-4478; hsa-miR-339-5p; hsa-miR-450a-3p; hsa-miR-185-3p

### MiRNAs-mRNAs integrative analysis reveals several hub-miRNAs in NPC development

**Figure 2:**
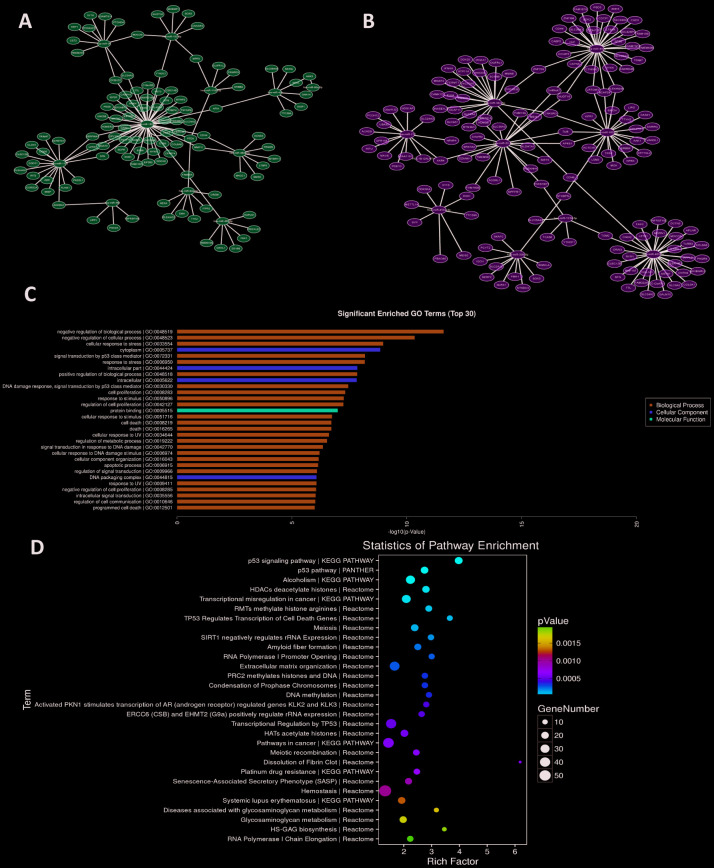
**MiRNAs-mRNAs regulatory networks.** (**A**, **B**) miRNAs-mRNAs network of up-/down-regulated miRNAs. (**C**, **D**) GO and pathway analyses of miR-182 target genes.

Based on NPC mRNAs expression data from GEO dataset (GSE126683), the Pearson Correlation between top 20 differentially expressed miRNAs and mRNAs was calculated. The miRNAs-mRNAs pairs with negative correlation were further screened by miRWalk3.0 database (Supplementary Table 1). As shown in Figure 2A, 2B, the up-/down-regulated miRNAs-mRNAs pairs were displayed. Several hub-miRNAs with more than fifteen target genes may play crucial roles during NPC pathogenesis (miR-182, miR-1183, miR-34c-5p, miR-4479, miR-185-3p and miR-455-3p), especially miR-182, which has the highest number of target genes.

### The high expression and potential function of miR-182 in NPC

**Figure 3:**
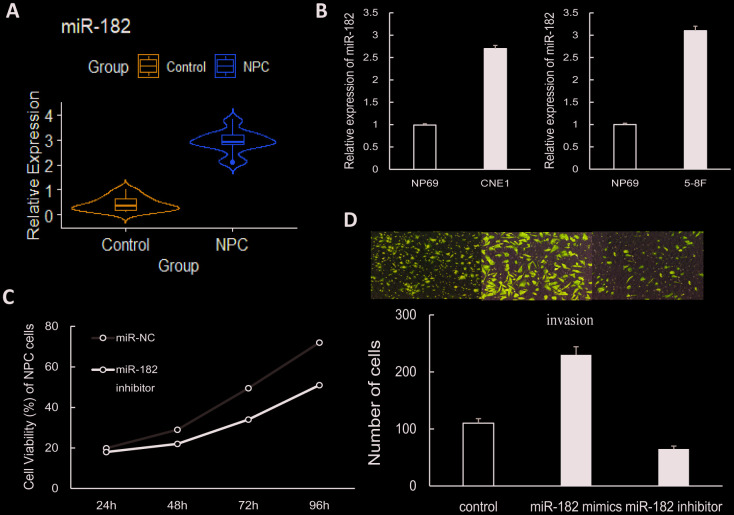
**The role of miR-182 in NPC cells.** (**A**) The violin plot shows that miR-182 was up-regulated in NPC (patient samples: n = 32; control samples: n = 20). (**B**) The expression of miR-182 was significantly increased in NPC cell lines (CNE1 and 5-8F). (**C**, **D**) Proliferation and invasion of NPC cells were significantly increased in miR-182 mimics group and decreased in miR-182 inhibitor group. The data presented here is the mean (SD) of triplicate replicates. *P* < 0.05.

To explore the role of miR-182 in NPC, the expression level of miR-182 was measured in 32 NPC patient samples and 20 control samples by qRT-PCR assay. We found that miR-182 expression was significantly increased in NPC group (Figure 3A). Validation of miR-182 expression in two NPC cell lines (CNE1 and 5-8F) showed consistent results (*P* < 0.01) (Figure 3B). To further explore the biological function of miR-182, the validated target genes were extracted from miRWalk3.0 database and further subjected for functional enrichment analysis (GO and KEGG). The significant enriched GO terms (*P* < 0.05) were obtained in Figure 2C, including negative regulation of biological process, signal transduction by p53 class mediator and regulation of cell proliferation. MiR-182 mediated pathways were mainly participated in p53 signaling pathway, transcriptional misregulation in cancer and DNA methylation (*P* < 0.05) (Figure 2D). Moreover, we examined the correlation between miR-182 expression and NPC clinical outcomes by Chi-square test. The results indicated that high expression of miR-182 was strongly correlated with TNM stage (P-value = 0.008) and lymph node migration (P-value = 0.002) in NPC patients (Table 2).

**Table 2:** Expression of miR-182 and clinical features of NPC patients.

**Clinical index**	**MiR-182 expression**		***P*-value**
	**High**	**Low**	
Age			0.631
<40	8	7	
≥40	10	7	
Gender			0.277
Male	13	10	
Female	5	4	
Smoking			0.605
Yes	7	5	
No	11	9	
TNM stage			0.008
I-II	6	8	
III-IV	12	6	
Lymph nodes metastasis			0.002
Positive	4	1	
negative	14	13	

### MiR-182 promotes proliferation and invasion of NPC cells

To further explore the function of miR-182 in NPC, we performed loss-/gain-of-function experiment in 5-8F cells to examine whether miR-182 could modulate metastasis of NPC cells. CCK-8 result showed that cell proliferation was significantly suppressed by miR-182 inhibitor (Figure 3C). In addition, invasion of NPC cells was significantly increased in miR-182 mimics condition and decreased in miR-182 inhibitor condition as transwell experiment detected (Figure 3D). Besides, cell apoptosis results indicated that apoptosis of NPC cells was significantly suppressed in miR-182 mimics condition and increased in miR-182 inhibitor condition (Supplementary Figure 1A). The above findings indicated that miR-182 may play an oncogenic role in NPC cells.

### MiR-182 promotes proliferation and invasion of NPC cells via targeting PTEN

**Figure 4:**
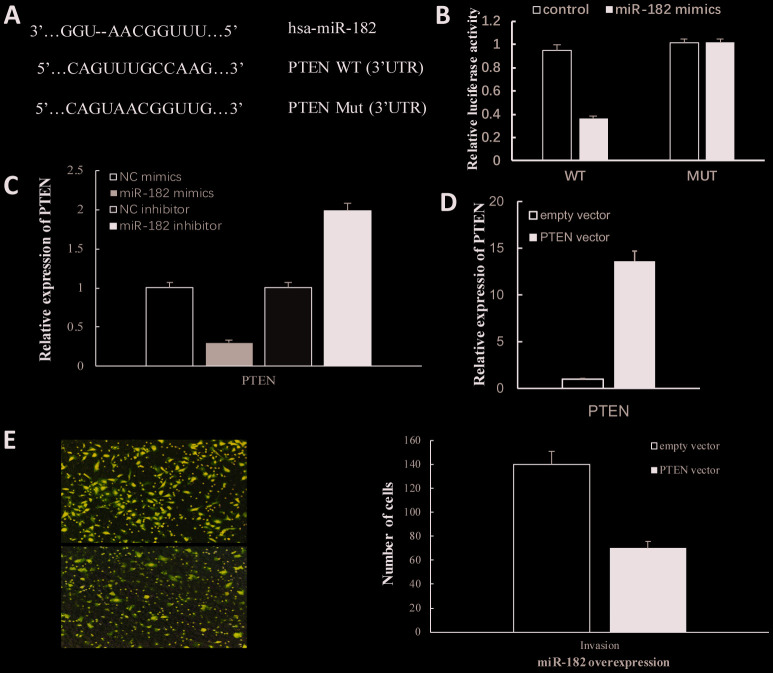
**MiR-182 promoted proliferation and invasion of NPC cells via targeting PTEN.** (**A**) The complementary binding sites of miR-182 and 3′UTR of PTEN. (**B**) The dual-luciferase reporter assays were performed to verify the target prediction. (**C**) MiR-182 mimics/inhibitors significantly reduced/increased PTEN expression. (**D**) PTEN expression was significantly increased after transfection. (**E**) Overexpression of PTEN abrogates the promotion effect of miR-182 mimics on NPC invasion. *P* < 0.05.

Bioinformatics algorithm revealed that 3’-UTR region of PTEN contains the binding sites of miR-182 (Figure 4A). As luciferase reporter assay showed that enhanced expression of miR-182 significantly decreased luciferase activity of PTEN wild type (*P* < 0.01) but made no difference in PTEN mutant (Figure 4B). In addition, we found that overexpression of miR-182 notably reduced expression of PTEN (*P* < 0.05) while suppressed expression of miR-182 notably increased PTEN expression (*P* < 0.05) (Figure 4C, Supplementary Figure 1B). After transfection of PTEN in 5-8F cells, the result showed that PTEN expression was notably increased (Figure 4D). Moreover, overexpression of PTEN could abrogate the promotion effect of miR-182 mimics on NPC invasion (*P* < 0.05) (Figure 4E). In addition, we inhibited PTEN expression with siRNA and found that PTEN-depleted cells showed increased proliferation. Moreover, transfection of miR-182 mimics into PTEN-depleted cells showed an increased proliferation of NPC cells compared to control (Supplementary Figure 1C). The above results demonstrated that miR-182 promotes NPC cells metastasis via targeting PTEN.

## DISCUSSION

As one of the most common malignant cancers in China, NPC is derived from epithelial cells and associated with complex biochemical, physiological and molecular processes [1, 2, 15]. In recent years, miRNAs are demonstrated as key regulators that participate in disease metastasis, which could be considered as novel therapeutic targets in cancer [7, 16]. So far, miRNAs in NPC has not been fully disclosed and integrative analysis of miRNAs and mRNAs remains largely unknown. MiR-182, Previous studies have shown that miR-182 plays an important role in tumor progression [17, 18], such as epithelial mesenchymal transition and lung cancer cells metastasis. However, the potential role of miR-182 in NPC remains largely unknown.

With the aid of RNA sequencing and bioinformatics analysis, 102 miRNAs were identified to be abnormally expressed in NPC samples, including 39 upregulated miRNAs and 63 downregulated miRNAs. These dysregulated miRNAs might serve as rich resources for NPC in clinical field. Several hub-miRNAs with more than fifteen target genes have been screened (miR-182, miR-1183, miR-34c-5p, miR-4479, miR-185-3p and miR-455-3p) and these miRNAs may play key roles during NPC pathogenesis. As one of the hub-miRNAs, miR-182 expression was notably increased in NPC tissues and cell lines compared to control. MiR-182 is one of miR-183 family, which is a cancer-related oncogenic miRNA and dysregulated in many clinical tissues [18]. For example, Oberbauer found that inhibition of miR-182 by ASO could improve kidney morphology after acute kidney injury [19]. MiR-182 could promote cancer invasion by linking RET oncogene activated NF-κB [20]. Functional enrichment analysis of miR-182 targets showed that miR-182 may involve in regulation of cell proliferation and p53 signaling pathway. By mining miRWalk3.0 database, PTEN was demonstrated to be a potential target of miR-182 and luciferase reporter assay confirmed this result. PTEN is a classical tumor suppressor in various cancers and acts as a crucial regulator in PI3K signaling pathway [13]. PTEN has been found to play key roles in NPC development, for example, BV-miR-BART7-3p can specifically target PTEN to regulate metastasis of nasopharyngeal carcinoma cells [21]. It’s worth mentioning that PTEN may not be the unique target of miR-182 and more experiments need to be conducted in the future. In this study, we found that overexpression of miR-182 remarkably suppressed PTEN expression while miR-182 inhibitor notably enhanced PTEN expression. Furthermore, the transwell experiment showed that overexpression of PTEN could abrogate the promotion effect of miR-182 on NPC invasion. These data indicated that up-regulation of miR-182 could promote proliferation and invasion of NPC cells via regulating PTEN expression.

In conclusion, the present study revealed miRNA expression profiles and miRNAs-mRNAs interaction pairs in NPC. The miRNA data obtained here are also important and essential to the next stage of NPC research. Moreover, we proved that up-regulation of miR-182 could promote proliferation and invasion of NPC cells via targeting PTEN.

## MATERIALS AND METHODS

### Clinical samples and cell culture

The human clinical samples were obtained from NPC patients (n = 32) before treatment and control samples (normal adjacent tissues, n = 20) (Supplementary Table 2). The experiment protocol was approved by institutional ethics committee and all of the participants and this study was in accordance with the Helsinki Declaration. NPC cells were isolated from NPC tissues and grown with 20% fetal bovine serum (Gibco, NY, USA). The nasopharyngeal epithelial cell line (NP69) and human NPC cell lines (5-8F and CNE1) were kindly provided by Liu J group (Central South University) and cultured in RPMI 1640 (Invitrogen, CA, USA).

### Illumina sequencing and miRNA expression analysis

Total RNA was extracted from four NPC tissues using Trizol and evaluated by denaturing agarose gel electrophoresis. After that, small RNA library was constructed and RNAs were with reverse transcribed using SuperScript II Reverse Transcriptase (Invitrogen, USA). The single-end sequencing was performed on Illumina HiSeq2500 platform (San Diego, CA, USA) according to the manufacturer’s protocol. The raw sequencing data of miRNA was filtered by FastQC and cutadapt software [22]. QC result was shown in Supplementary Table 3. MiRNAs were identified by miRDeep2 and the expression was normalized to transcripts per million (TPM). The differentially expressed miRNAs were calculated by R package DESeq2 [23] and setting the threshold as |Fold Change| > 1.5 and *P* < 0.01.

### Quantitative real-time PCR (qRT-PCR) experiment

Total RNA was isolated from tissue samples or cells using Trizol, then complementary DNA (cDNA) was obtained using TaqMan MiRNA Reverse Transcription kit (ThermoFisher, Waltham, MA). The primers were synthesized and designed by Biotech Company (Biotech, Shanghai, China). MiR-182: (forward) 5′-TTTACGCGTGTTGTTGTTGAGACAGAATCTCGCT-3′ and (reverse) 5′-TTTAAGCTTCCTGCCGACCCTGCGGAGAGA-3′; PTEN: (forward) 5′-AATTCCCAGTCAGAGGCGCTATGT-3′ and (reverse) 5′-GATTGCAAGTTCCGCCACTGAACA-3′). The qRT-PCR experiment was conducted with SYBR Green qPCR Mix (Fermentas) according to the instruction. The expression of miRNAs was normalized to U6. GAPDH was used to normalize the expression of mRNAs.

### Target prediction of miR-182 and dual-luciferase reporter assay

The dual-luciferase reporter assay was conducted to verify whether 3’UTR of PTEN contains the binding sites of miR-182. First, binding sites in 3’UTR of PTEN was cloned into a pGL3 Basic luciferase vector to obtain PTEN wild type plasmid (WT) or mutant type plasmid (Mut). After that, the above vectors were transfected into 5-8F NPC cell lines for 48 hours. Finally, the luciferase activity was normalized and then measured by a Luciferase Assay Kit (Promega).

### Functional enrichment of miR-182 target genes

Functional enrichment analysis (GO and KEGG pathway) of miR-182 target genes were analyzed by KOBAS software [24] which using a hypergeometric test. *P*-value < 0.01 and FDR < 0.05 were considered as significantly enriched.

### Cell transfection and apoptosis assay

The negative control vectors, miR-182 mimics and inhibitors were synthesized by Biotech Company (Biotech, Beijing, China). The NPC 5-8F cells were seeded for 24 hours and then transfected using Lipofectamine 2000 (Invitrogen, CA, USA) following the manufacturer’s instructions. Cell apoptosis assay was performed after transfection. Then an Annexin V-fluorescein isothiocyanate (BD Biosciences) and propidium iodide (BD Biosciences) were used to incubate cells (30 min). The apoptosis was measured by flow cytometry.

### Transwell experiment and Cell counting kit-8 (CCK-8) assay

The transwell experiment was conducted to study the invasive response of NPC 5-8F cells. The transfected cells were assayed using transwell chambers (EMD, MA, USA) and placed into 24-well plate with 8 μm BioCoat (BD, MA, USA) at 37°C with 5% CO2 for 24 hours. Optical-microscope (200 ×) was used to count of cell numbers in randomly selected fields. For CCK-8 experiment, the cells were seeded in a 96-well plate (1×10^3^/well) and further treated with CCK-8 solution (10 μL) according to the manufacturer’s protocol. The absorbance at 450 nM was observed. The experiments were performed independently for three times.

### Statistical analysis

Statistical analysis in this study was conducted using R software (https://www.r-project.org/). The Student’s T-test was used for difference analyses and setting the threshold as *P* < 0.05.

## Supplementary Material

Supplementary Figure 1

Supplementary Table 1

Supplementary Tables 2 and 3
