# Supplementary material for: Integrative analysis of miRNAs-mRNAs reveals that miR-182 up-regulation contributes to proliferation and invasion of nasopharyngeal carcinoma by targeting PTEN
Source: Aging (Albany NY). 2020 Jun 15;12(12):11568–78. doi: 10.18632/aging.103316 (PMC7343470; doi:10.18632/aging.103316)
Supplement: Supplementary Figure 1 [file aging-12-103316-s003..pdf]

SUPPLEMENTARY FIGURE

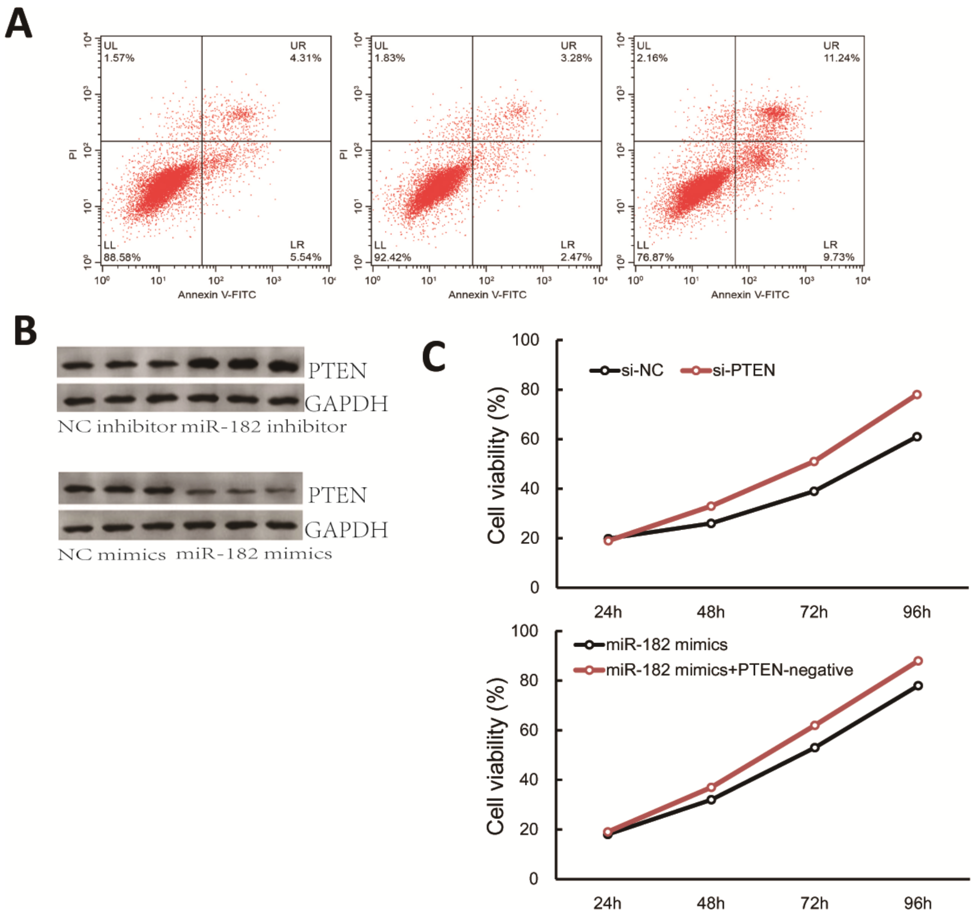

Supplementary Figure 1. Molecular experiments. (**A**) Cell apoptosis results indicated that apoptosis of NPC cells was significantly suppressed in miR-182 mimics condition and increased in miR-182 inhibitor condition; (**B**) Overexpression of miR-182 notably reduced expression of PTEN ($P < 0.05$) while suppressed expression of miR-182 notably increased PTEN expression ($P < 0.05$); (**C**) Transfection of miR-182 mimics into PTEN- depleted cells showed an increased proliferation of NPC cells compared to control.
