# Supplementary material for: Integrative analysis of miRNAs-mRNAs reveals that miR-182 up-regulation contributes to proliferation and invasion of nasopharyngeal carcinoma by targeting PTEN
Source: Aging (Albany NY). 2020 Jun 15;12(12):11568–78. doi: 10.18632/aging.103316 (PMC7343470; doi:10.18632/aging.103316)
Supplement: Supplementary Tables 2 and 3 [file aging-12-103316-s002..pdf]

## SUPPLEMENTARY TABLES

**Supplementary Table 2. Clinical information of NPC patients.**

| Characteristics | |
|---|---|
| Age, years | 44.98±11.86 |
| BMI | 21±5.25 |
| Gender | |
| Male, n (%) | 73.5% |
| Female, n (%) | 26.5% |
| T stage | |
| T1 | 23.7% |
| T2 | 22.9% |
| T3 | 24.9% |
| T4 | 28.5% |
| N stage | |
| N0 | 9.0% |
| N1 | 31.5% |
| N2 | 46.3% |
| N3 | 13.2% |

**Supplementary Table 3. Sample informations.**

| Sample | Num. of Raw Reads | Clean Reads% | RemoveAdapter% | InsertNull% | N% | too_short% | Poly-A% | too_long% | low_quality% |
|---|---|---|---|---|---|---|---|---|---|
| NPC1 | 11157702 | 89.43% | 0.03% | 0.42% | 0.36% | 5.37% | 0.06% | 3.15% | 1.19% |
| NPC2 | 11207287 | 90.50% | 0.05% | 0.29% | 0.36% | 4.78% | 0.14% | 2.77% | 1.11% |
| NPC3 | 10937374 | 88.13% | 0.04% | 0.51% | 0.03% | 6.80% | 0.10% | 3.82% | 0.56% |
| NPC4 | 10197246 | 92.34% | 0.03% | 0.27% | 0.04% | 3.55% | 0.09% | 2.99% | 0.70% |
| Control1 | 10956842 | 94.61% | 0.03% | 0.40% | 0.03% | 2.25% | 0.09% | 2.11% | 0.48% |
| Control2 | 12011555 | 85.54% | 0.05% | 0.53% | 0.03% | 6.51% | 0.10% | 6.52% | 0.72% |
| Control3 | 12203144 | 88.40% | 0.04% | 0.38% | 0.13% | 3.90% | 0.13% | 6.23% | 0.79% |
| Control4 | 11356664 | 87.88% | 0.04% | 0.28% | 0.03% | 3.74% | 0.15% | 7.25% | 0.62% |
